# Congruency, Homomorphism  and Isomorphism on Autometrized Algebras

**DOI:** 10.12688/f1000research.159591.1

**Published:** 2025-01-03

**Authors:** Gebrie Yeshiwas Tilahun

**Affiliations:** 1 Department of Mathematics, Assosa University, Asosa, Benishangul-Gumuz, Ethiopia

**Keywords:** autometrized algebra, normal autometrized algebra, equivalent relation, congruence, homomorphism, isomorphism

## Abstract

This paper presents a study of congruence relations on autometrized algebras. We demonstrate that in a normal autometrized algebra, which satisfies certain conditions, the set of all congruence relations forms a complete sublattice within the set of all equivalence relations. Furthermore, we investigate the property that a congruence-permutable autometrized algebra is also congruence-modular. We also explore several fundamental properties related to congruence relations. Additionally, we introduce the kernel of a homomorphism and establish that it is a congruence relation. Lastly, we examine the homomorphism, isomorphism, and correspondence theorems of autometrized algebra using the concept of congruence.

## 1. Introduction

The concept of autometrized algebras was first introduced by K. N. Swamy (1964) to develop a unified theory containing previously established autometrized algebras, including Boolean algebras ( Blumenthal 1952; Ellis 1951), Brouwerian algebras ( Nordhaus & Lapidus 1954), Newman algebras ( Roy 1960), autometrized lattices ( Nordhaus & Lapidus 1954), and commutative lattice-ordered groups, or l-groups ( Narasimha Swamy 1964). The study of congruences within autometrized algebras was introduced by K. Swamy & Rao (1977). Subsequent developments in the theory of autometrized algebras were contributed by K. Swamy & Rao (1977), Rachŭnek (1987, 1989, 1990, 1998), Hansen (1994), Kovář (2000), and Chajda & Rachunek (2001).

Furthermore, the notion of representable autometrized algebras was explored by Subba Rao & Yedlapalli (2018) and later by Rao et al. (2019, 2021, 2022). In their research, Tilahun et al. (2023a, b, c, d) established a theory concerning subalgebras, ideals, and homomorphisms. They analyzed the relationship between normal autometrized l-algebras and representable autometrized algebras. Additionally, they introduced the concepts of direct and subdirect products of autometrized algebras, convex subalgebras, and congruences within autometrized algebras. However, previous studies have not explored the properties and applications of congruence relations in autometrized algebras. Therefore, our motivation is to address these gaps. This paper presents the concept of congruency in autometrized algebras, along with the introduction and proof of various theorems and facts related to congruence relations. The study will also cover homomorphisms, isomorphisms, and correspondence theorems for autometrized algebras through the concept of congruency.

The structure of this paper is as follows: Section 2 will provide definitions and terminology. Section 3 will introduce the concept of congruency in autometrized algebras. Section 4 will present theorems related to homomorphisms and isomorphisms in autometrized algebras. Finally, Section 5 will conclude the paper.

## 2. Preliminaries

In this section, we’ll look at some essential concepts, definitions, and terms; that are important in other sections.

Definition 2.1 ( K. N. Swamy 1964) *A system A=* (A,+,0,≤,∗) *is called an autometrized algebra if*

(i)
(A,+,0) *is a commutative monoid.*
(ii)
(A,≤) *is a partial ordered set, and* ≤ *is translation invariant, that is,* ∀a,b,c∈A;a≤b⇒a+c≤b+c .
(iii)
∗:A×A→A *is autometric on* A *, that is,* ∗ *satisfies metric operation axioms:*
( *M*_1_) ∀a,b∈A;a∗b≥0 *and,* a∗b=0⇔a=b ,
( *M*_2_) ∀a,b∈A;a∗b=b∗a ,
( *M*_3_) ∀a,b,c∈A *;* a∗c≤a∗b+b∗c .

Definition 2.2 ( K. Swamy & Rao 1977) *An autometrized algebra A =* (A,+,0,≤,∗) *is called normal if and only if*

(i)
a≤a∗0∀a∈A.

(ii)
(a+c)∗(b+d)≤(a∗b)+(c∗d)∀a,b,c,d∈A.

(iii)
(a∗c)∗(b∗d)≤(a∗b)+(c∗d)∀a,b,c,d∈A.

(iv) *For any* a *and* b *in A,* a≤b⇒∃x≥0 *such that* a+x=b .

Definition 2.3 ( K. Swamy & Rao 1977) *Let* A=(A,+,0,≤,∗) *be a system. Then* A *is said to be a lattice ordered autometrized algebra (or) autometrized l-algebra if*

(i)
(A,+,0) *is a commutative semigroup with 0.*
(ii)
(A,≤) *is a lattice, and* ≤ *is translation invariant, that is,* ∀a,b,c∈A *;*
$$\begin{array}{l} a+\left(b\vee c\right)=\left(a+b\right)\vee \left(a+c\right)\\ a+\left(b\wedge c\right)=\left(a+b\right)\wedge \left(a+c\right) \end{array}$$
(iii)
∗:A×A→A *is autometric on* A *, that is,* ∗ *satisfies metric operation axioms:* $M_{1}$ *,* $M_{2}$ *and* $M_{3}$ .

Definition 2.4 ( Tilahun et al. 2023d) *Let A =* (A,+,0,≤,∗) *be autometrized algebra. Let* B⊆A *. Then* B *is said to be a subalgebra of* A *if;*

(i)
(B,+,0) *is a commutative monoid.*
(ii)
(B,≤) *is a subposet, and* ≤ *is translation invariant, that is,* a≤b⇒a+c≤b+c *for any* a,b,c∈B .
(iii)
∗|B:B×B→B *is metric.
*

Definition 2.5 ( Tilahun et al. 2023d) *A nonempty subset* I *of an autometrized algebra A =* (A,+,0,≤,∗) *is called an ideal if and only if*

(i)
a,b∈I *imply* a+b∈I .
(ii)
a∈I,b∈Aandb∗0≤a∗0 *imply* b∈I .

Definition 2.6 ( K. Swamy & Rao 1977) *Let* A *be an autometrized algebra. Then* A *is said to be semiregular if for any* a∈A *,* a≥0⇒a∗0=a .

Definition 2.7 ( K. Swamy & Rao 1977) *Let* A *be a normal autometrized algebra. An equivalence relation* Θ *on* A *is called a congruence relation if*

(i)
(a,b),(c,d)∈Θ⇒(a+c,b+d)∈Θ,∀a,b,c,d∈A,

(ii)
(a,b),(c,d)∈Θ⇒(a∗c,b∗d)∈Θ,∀a,b,c,d∈A,

(iii)
(a,b)∈Θandx∗y≤a∗b⇒(x,y)∈Θ,∀a,b,x,y∈A.

Definition 2.8 ( K. Swamy & Rao 1977) *Let* A *be a normal autometrized algebra and* Θ *be a congruence on* A *. Then,* A/Θ *= the set of all equivalence classes of* Θ *in* $A=\left\{\bar{x}=\Theta \left(x\right)|x\in A\right\}$ .

Theorem 2.9 ( K. Swamy & Rao 1977) *Let* A *be a normal autometrized algebra. Let* Θ *be a congruence relation on* A *. For any* $\bar{a},\;\bar{b}\in A/\Theta$ *, Define:*

$$\begin{array}{l} \bar{a}+\bar{b}=\bar{a+b}.\\ \bar{a}*\bar{b}=\bar{a*b}.\\ \bar{a}\leq \bar{b}\Leftrightarrow \exists x\geq 0\;\text{such}\;\text{that}\;\left(a+x,\;b\right)\in \Theta . \end{array}$$

*Then* (A/Θ,+,≤,∗) *is a normal autometrized algebra if and only if* Θ *has the following property: For any* a,b∈A *and* $z_{1}\geq 0,\;z_{2}\geq 0$ *;*

$$\left(a+z_{1},\;b\right)\in \Theta \;\text{and}\;\left(b+z_{2},\;a\right)\in \Theta \Rightarrow \left(a,\;b\right)\in \Theta .$$

Theorem 2.10 ( K. Swamy & Rao 1977) *Let* A *be normal autometrized algebra. Let* ℐ(A) *= set of all ideals of* A *. Let* Con(A) *= set of all congruences on* A *. Then* ℐ(A) *and* Con(A) *are in one-to-one correspondence.
*

Definition 2.11 ( Tilahun et al. 2023d) *Let A =* (A,+,0,≤,∗) *and B =* (B,+,0,≤,∗) *be autometrized algebras. Let* f:A→B *be a map. Then* f *is said to be a homomorphism from* A *to* B *if and only if*

(i)
f(a+b)=f(a)+f(b)∀a,b∈A,

(ii)
f(a∗b)=f(a)∗f(b)∀a,b∈Aand

(iii)
a≤b⇒f(a)≤f(b)∀a,b∈A.

A homomorphism f:A→B is called

(i) an epimorphism if and only if f is onto.
(ii) a monomorphism(embedding) if and only if f is one-to-one.
(iii) an isomorphism if and only if f is a bijection.

Definition 2.12 ( Tilahun et al. 2023d) *Let* A,B *be normal autometrized algebras. Let* f:A→B *be a homomorphism. Then* $\ker f=\left\{x\in A|\;f\left(x\right)=\bar{0}\right\}$ *where* $\bar{0}$ *is the zero element of* B .

*Clearly,* f *is one-to-one if and only if* kerf={0} .

Definition 2.13 *Let* A *and* B *be autometrized algebras. Let* f:A→B *be a map. If* a≤b⇔f(a)≤f(b),∀a,b∈A *, then* f *is said to be an order-embedding of* A *in to* B *. That is,* f *is both order-preserving and order-reversing.
*

Remark 2.14 *If* f *is an order-embedding of* A *in to* B *, then* f *is necessary injective. Since* f(a)=f(b) *implies* a≤b *and* b≤a *and in turn* a=b *according to antisymmetry of* ≤ .

Theorem 2.15 ( K. Swamy & Rao 1977) *Let* A *be a normal autometrized algebra. Then every ideal of* A *is a kernel of an epimorphism of* A *. That is;*

(i) *Kernel of any epimorphism of* A *is an ideal of* A .
(ii) *If* I *is an ideal of* A *, then there exists an epimorphism* f *on* A *such that* kerf=I .

## 3. Congruency on autometrized algebra

In this section, we demonstrate that in a normal autometrized algebra, which satisfies certain conditions, the set of all congruence relations forms a complete sublattice within the set of all equivalence relations. We prove that a congruence-permutable autometrized algebra is also congruence-modular.

Definition 3.1 *Let* A *be an autometrized algebra. Let* Θ *be a relation on* A *. Then* Θ *is said to be satisfied compatibility property, if*

(i)
(a,b),(c,d)∈Θ⇒(a+c,b+d)∈Θ∀a,b,c,d∈A,

(ii)
(a,b),(c,d)∈Θ⇒(a∗c,b∗d)∈Θ∀a,b,c,d∈A,

(iii)
(a,b)∈Θ *and* x∗y≤a∗b⇒(x,y)∈Θ∀a,b,x,y∈A .

Definition 3.2 *Let* A *be an autometrized algebra. The set of all equivalence relations on* A *is denoted by* Eq(A) .

Definition 3.3 *Let* A *be an autometrized algebra. Let* Θ∈Eq(A) *. Then* Θ *is said to be a congruence on* A *if it satisfies compatibility property.*

Definition 3.4 *Let* A *be an autometrized algebra. The set of all congruence relations on* A *is denoted by* Con(A) .

Definition 3.5 *Let* A *be an autometrized algebra. Then* Δ={(a,b)∈A×A|a=b}={(a,a)|a∈A} *is called diagonal relation on* A *. And also* ∇=A×A *is called all relation on* A .

Example 3.6 *Let* A={0,a,b,c} *with* 0≤a≤b≤c *. Define* ∗ *and* + *by the following tables.*

**Table:** 

∗	0	a	b	c
0	0	a	b	c
a	a	0	b	c
b	b	b	0	c
c	c	c	c	0

**Table:** 

+	0	a	b	c
0	0	a	b	c
a	a	a	b	c
b	b	b	b	c
c	c	c	c	c

*It is clear to show that* A *is an autometrized algebra. Let us consider the equivalent relations* Δ={(0,0),(a,a),(b,b),(c,c)} *,* $\Theta_{1}=\left\{(0,\;0),\;\left(a,\;a\right),\;\left(b,\;b\right),\;\left(c,\;c\right),\;\left(0,\;a\right),\;\left(a,\;0\right)\right\}$ *,* $\Theta_{2}=\left\{(0,\;0),\;\left(a,\;a\right),\;\left(b,\;b\right),\;\left(c,\;c\right),\;\left(a,\;0\right),\;\left(0,\;a\right),\;\left(0,\;b\right),\;\left(b,\;0\right),\;\left(a,\;b\right),\;\left(b,\;a\right)\right\}$ *and* ∇=A×A *on* A *. Then* Δ *,* $\Theta_{1}$ *,* $\Theta_{2}$ *and* ∇ *are all the congruence relations on* A *. Therefore,* $\mathrm{Con}\left(A\right)=\left\{\Delta ,\;\Theta_{1},\;\Theta_{2},\;\nabla \right\}$ .

Definition 3.7 *Let* A *be an autometrized algebra and* X⊆A *. Then the smallest congruence containing* X×X *is called the congruence generated by* X×X *or* X *and denoted by* Θ(X×X) *or* Θ(X) *. Further, if* X={a,b} *, then* Θ(X) *=* Θ(a,b) *is called principal congruence.
*

Example 3.8 *In* *Example* (3.6) *,* A *is an autometrized algebra. Take* X={a,b} *. Then,* X×X={(a,a),(b,b),(a,b),(b,a)} *. Therefore,* Θ(X×X)=Θ(X)={(0,0),(a,a),(b,b),(c,c),(a,0),(0,a),(0,b),(b,0),(a,b),(b,a)} *. Hence,* Θ(X) *is a principal congruence.
*

Definition 3.9 *Let* $\Theta_{1}$ *,* $\Theta_{2}$ *be relations on an autometrized algebra* A *. Then their product is denoted by* $\Theta_{1}◦\Theta_{2}$ *, defined as:*

$$\left(a,\;b\right)\in \Theta_{1}◦\Theta_{2}\Leftrightarrow \exists c\in A\;\text{such}\;\text{that}\left(a,\;c\right)\in \Theta_{1}\;\text{and}\;\left(c,\;b\right)\in \Theta_{2}.$$

*That is;*

$$\Theta_{1}◦\Theta_{2}=\left\{(a,\;b)|\exists c\in A\;\text{such}\;\text{that}\;\left(a,\;c\right)\in \Theta_{1}\;\text{and}\;\left(c,\;b\right)\in \Theta_{2}\right\}.$$

Example 3.10 *It is clear that in Example* (3.6) *,* A *is an autometrized algebra. And we know that* $\Theta_{1}=\left\{(0,\;0),\;\left(a,\;a\right),\;\left(b,\;b\right),\;\left(c,\;c\right),\;\left(0,\;a\right),\;\left(a,\;0\right)\right\}$ *,* $\Theta_{2}=\left\{(0,\;0),\;\left(a,\;a\right),\;\left(b,\;b\right),\;\left(c,\;c\right),\;\left(a,\;0\right),\;\left(0,\;a\right),(0,\;b),\;\left(b,\;0\right),\;\left(a,\;b\right),\;\left(b,\;a\right)\right\}$ *. Therefore,* $\Theta_{1}◦\Theta_{2}=\left\{(0,\;0),\;\left(a,\;a\right),\;\left(b,\;b\right),\;\left(c,\;c\right),\;\left(a,\;0\right),\;\left(0,\;a\right),(0,\;b),\;\left(b,\;0\right),\;\left(a,\;b\right),\;\left(b,\;a\right)\right\}$ .

Remark 3.11 *If* $\Theta_{1},\;\Theta_{2}\in \mathrm{Con}\left(A\right)$ *, then* $\Theta_{1}◦\Theta_{2}$ *need not be a congruence relation on* A .

Theorem 3.12 *If* $\Theta_{1}$ *,* $\Theta_{2}$ *are two congruence relations on a normal autometrized algebra* A *satisfying* a≤b⇒a∗c≤b∗c∀a,b,c∈A *and* b≠c *, then* $\Theta_{1}◦\Theta_{2}$ *satisfies compatibility property.
*

Proof.
Now to show that $\Theta_{1}◦\Theta_{2}$ satisfies compatibility property.

(i) Suppose $\left(a,\;b\right),\;\left(c,\;d\right)\in \Theta_{1}◦\Theta_{2}$ . There exist x,y∈A such that $\left(a,\;x\right)\in \Theta_{1}$ , $\left(x,\;b\right)\in \Theta_{2}$ and $\left(c,\;y\right)\in \Theta_{1}$ , $\left(y,\;d\right)\in \Theta_{2}$ . Since $\Theta_{1}$ and $\Theta_{2}$ are congruence relations; $\left(a+c,\;x+y\right)\in \Theta_{1}$ and $\left(x+y,\;b+d\right)\in \Theta_{2}$ . Thus, $\left(a+c,\;c+d\right)\in \Theta_{1}◦\Theta_{2}$ .
(ii) Suppose $\left(a,\;b\right),\;\left(c,\;d\right)\in \Theta_{1}◦\Theta_{2}$ . There exist x,y∈A such that $\left(a,\;x\right)\in \Theta_{1}$ , $\left(x,\;b\right)\in \Theta_{2}$ and $\left(c,\;y\right)\in \Theta_{1}$ , $\left(y,\;d\right)\in \Theta_{2}$ . Since $\Theta_{1}$ and $\Theta_{2}$ are congruence relations; $\left(a*c,\;x*y\right)\in \Theta_{1}$ and $\left(x*y,\;b*d\right)\in \Theta_{2}$ . Thus, $\left(a*c,\;c*d\right)\in \Theta_{1}◦\Theta_{2}$ .
(iii) Suppose $\left(a,\;b\right)\in \Theta_{1}◦\Theta_{2}$ and x∗y≤a∗b . To show that $\left(x,\;y\right)\in \Theta_{1}◦\Theta_{2}$ . There exists z∈A such that $\left(a,\;z\right)\in \Theta_{1}$ and $\left(z,\;b\right)\in \Theta_{2}$ . It clear that
x∗y≤a∗b≤a∗z+b∗z. (1)
Since $\Theta_{1}$ is a congruence relation; $\left(z,\;z\right),\;\left(b,\;b\right)\in \Theta_{1}$ . As a result, $\left(a*z,\;0\right),\;\left(a*b,\;b*z\right)\in \Theta_{1}$ . This implies that $\left(a*z+b*z,\;a*b\right)\in \Theta_{1}$ . We know that 0≤a∗b . By the given condition (a∗z+b∗z)∗0≤(a∗z+b∗z)∗(a∗b) . Since A is normal; equation (1) becomes
x∗y≤a∗b≤a∗z+b∗z≤(a∗z+b∗z)∗0≤(a∗z+b∗z)∗(a∗b).
Since $\left(a*z+b*z,\;a*b\right)\in \Theta_{1}$ and x∗y≤(a∗z+b∗z)∗(a∗b) ; by definition of congruence $\left(x,\;y\right)\in \Theta_{1}$ . It is clear that $\left(y,\;y\right)\in \Theta_{2}$ . Therefore, $\left(x,\;y\right)\in \Theta_{1}◦\Theta_{2}$ . Hence, $\Theta_{1}◦\Theta_{2}$ satisfies compatibility property.

Theorem 3.13 *Let* Θ _1_, Θ _2_ *be two congruence relations on a normal autometrized algebra A satisfying a* ≤ *b* ⇒ *a* ∗ *c* ≤ *b* ∗ *c* ∀ *a, b, c* ∈ *A and b ≠ c. Suppose that* Θ _1_ ◦ Θ _2_ = Θ _2_ ◦ Θ _1_ *. Then* Θ _1_ ◦ Θ _2_ *is a congruence relation on A.
*

Proof.
To show that the product Θ _1_ ◦ Θ _2_ is an equivalence relation.

(i) *Reflexive*: Let *x* ∈ *A.* We know that ( *x*, *x*) ∈ Θ _1_ and ( *x*, *x*) ∈ Θ _2_. Therefore ( *x*, *x*) ∈ Θ _1_ ◦ Θ _2_ ∀ *_x_* ∈ *A.*
(ii) *Symmetric*: Suppose ( *x*, *y*) ∈ Θ _1_ ◦ Θ _2_. There exists *z* ∈ *A* such that ( *x*, *z*) ∈ Θ _1_ and ( *z*, *y*) ∈ Θ _2_. Since Θ _1_ and Θ _2_ are equivalence relations; ( *z*, *x*) ∈ Θ _1_ and ( *y*, *z*) ∈ Θ _2_. As a result, ( *y*, *x*) ∈ Θ _2_ ◦ Θ _1_. Since Θ _1_ ◦ Θ _2_ = Θ _2_ ◦ Θ _1_; and hence ( *y*, *x*) ∈ Θ _1_ ◦ Θ _2_.
(iii) *Transitivity*: Suppose ( *x*, *y*), ( *y*, *z*) ∈ Θ _1_ ◦ Θ _2_. There exist *r*, *q* ∈ *A* such that ( *x*, *r*) ∈ Θ _1_, ( *r*, *y*) ∈ Θ _2_ and ( *y*, *q*) ∈ Θ _1_, ( *q*, *z*) ∈ Θ _2_. Consequently, ( *r*, *q*) ∈ Θ _2_ ◦ Θ _1_, ( *r*, *z*) ∈ (Θ _2_ ◦ Θ _1_) ◦ Θ _2_ and ( *x*, *z*) ∈ Θ _2_ ◦ (Θ _2_ ◦ Θ _1_) ◦ Θ _2_. Since Θ _1_ ◦ Θ _2_ = Θ _2_ ◦ Θ _1_; ( *x*, *z*) ∈ Θ _2_ ◦ (Θ _1_ ◦ Θ _2_) ◦ Θ _2_. This implies that there exist *f*, *g* ∈ *A* such that ( *x*, *f* ) ∈ Θ _1_, ( *f*, *g*) ∈ Θ _1_ ◦ Θ _2_ and ( *g*, *z*) ∈ Θ _2_. Since ( *f*, *g*) ∈ Θ _1_ ◦ Θ _2_; there exists *l* ∈ *A* such that ( *f*, *l* ) ∈ Θ _1_ and ( *l*, *g*) ∈ Θ _2_. Since Θ _1_ and Θ _2_ are equivalence relations; ( *x*, *l* ) ∈ Θ _1_ and ( *l*, *z*) ∈ Θ _2_. Hence ( *x*, *z*) ∈ Θ _1_ ◦ Θ _2_. Hence, Θ _1_ ◦ Θ _2_ is an equivalence relation on *A.*

By theorem (3.12); we know that Θ _1_ ◦ Θ _2_ satisfies compatibility property. Hence, Θ _1_ ◦ Θ _2_ is a congruence relation on *A.
*

Example 3.14 *It is clear that in Example* (3.6), *A is a normal autometrized algebra satisfying a* ≤ *b* ⇒ *a* ∗ *c* ≤ *b* ∗ *c* ∀ *a*, *b*, *c* ∈ *A and b* ≠ *c.* *And we know that* Θ _1_ = {(0, 0), ( *a*, *a*), ( *b*, *b*), ( *c*, *c*), (0, *a*), ( *a*, 0)}, Θ _2_ = {(0, 0), ( *a*, *a*), ( *b*, *b*), ( *c*, *c*), ( *a*, 0), (0, *a*), (0, *b*), ( *b*, 0), ( *a*, *b*), ( *b*, *a*)}. Therefore, Θ _1_ ◦ Θ _2_ = Θ _2_ ◦ Θ _1_ = Θ _2_. *Hence,* Θ _1_ ◦ Θ _2_ *is a congruence relation on A.
*

Theorem 3.15 *Let A be a normal autometrized algebra A satisfying a* ≤ *b* ⇒ *a* ∗ *c* ≤ *b* ∗ *c* ∀ *a*, *b*, *c* ∈ *A and b* ≠ *c. Then* ( *Con*( *A*), ⊆) *is a complete sublattice of* ( *Eq*( *A*), ⊆).

Proof.
Let {Θ *_i_*} _*i*∈ *I*_ ⊆ *Con*( *A*). Therefore, {Θ *_i_*} _*i*∈ *I*_ ⊆ *Eq*( *A*). We know that

$$\begin{array}{l} {\mathrm{Inf}}_{\mathrm{Eq}\left(A\right)}{\left\{\Theta_{i}\right\}}_{i\in I}=\underset{i\in I}{\wedge }\Theta_{i}=\cap_{i\in I}\Theta_{i}.\\ {\mathrm{Sup}}_{\mathrm{Eq}\left(A\right)}{\left\{\Theta_{i}\right\}}_{i\in I}=\underset{i\in I}{\vee }\Theta_{i}=\cup \left\{\Theta_{0}◦\Theta_{1}◦\cdots ◦\Theta_{k}|0,\;1,\cdots k\in I\right\}. \end{array}$$

Clearly, $\cap_{i\in I}\Theta_{i}\in \mathrm{Con}\left(A\right)$ . So, ${\mathrm{Inf}}_{\mathrm{Eq}\left(A\right)}{\left\{\Theta_{i}\right\}}_{i\in I}=\wedge_{i\in I}\Theta_{i}\in \mathrm{Con}\left(A\right)$ .

To show that ${\mathrm{Sup}}_{\mathrm{Eq}\left(A\right)}{\left\{\Theta_{i}\right\}}_{i\in I}=\vee_{i\in I}\Theta_{i}\in \mathrm{Con}\left(A\right)$ . Let $\Psi =\cup \left\{\Theta_{0}◦\Theta_{1}◦\cdots ◦\Theta_{k}|0,\;1,\cdots k\in I\right\}$ . To show that Ψ∈Con(A) . Clearly, Ψ∈Eq(A) .

To show that Ψ satisfies compatibility property. Let (a,b)∈Ψ and x∗y≤a∗b . Therefore, $\left(a,\;b\right)\in \Theta_{0}◦\Theta_{1}◦\cdots ◦\Theta_{i}$ for i=0,⋯,k . By theorem (3.12); $\left(x,\;y\right)\in \Theta_{0}◦\Theta_{1}◦\cdots ◦\Theta_{i}$ . It is clear that (x,y)∈Ψ . Therefore, ${\mathrm{Sup}}_{\mathrm{Eq}\left(A\right)}{\left\{\Theta_{i}\right\}}_{i\in I}=\vee_{i\in I}\Theta_{i}\in \mathrm{Con}\left(A\right)$ . Hence, Con(A) is a complete sublattice of Eq(A) .

Definition 3.16 *Let A be an autometrized algebra and θ, φ* ∈ *Con*( *A*) *. Then θ, φ are said to be permutable congruences if θ* ◦ *φ = φ* ◦ *θ.
*

Example 3.17 *It is clear that in Example* (3.6) *;* $\Theta_{1},\;\Theta_{2}$ *are permutable congruences. Since* $\Theta_{1}◦\Theta_{2}=\Theta_{2}◦\Theta_{1}=\Theta_{2}$ .

Definition 3.18 *Let* A *be an autometrized algebra. If every pair of elements in* Con(A) *are permutable, then* A *is said to be a congruence-permutable autometrized algebra.
*

Example 3.19 *It is clear that in Example* (3.6) *;* $\mathrm{Con}\left(A\right)=\left\{\Delta ,\;\Theta_{1},\;\Theta_{2},\;\nabla \right\}$ *. Further,* $\Delta ◦\Theta_{1}=\Theta_{1}◦\Delta ,\;\Delta ◦\Theta_{2}=\Theta_{2}◦\Delta ,\;\Delta ◦\nabla =\nabla ◦\Delta ,\;\Theta_{1}◦\Theta_{2}=\Theta_{2}◦\Theta_{1},\;\Theta_{1}◦\Delta =\Delta ◦\Theta_{1}$ *, and* $\Theta_{2}◦\Delta =\Delta ◦\Theta_{2}$ *. Hence,* A *is a congruence-permutable autometrized algebra.
*

Definition 3.20 *Let* A *be an autometrized algebra. Then* A *is said to be a congruence-modular algebra if* Con(A) *is a modular lattice.
*

Theorem 3.21 ( Burris 1981) *Let* A *be an autometrized algebra and* θ,ϕ∈Eq(A) *. Then the following are equivalent.*

(i)
θ◦ϕ=ϕ◦θ.

(ii)
θ∨ϕ=θ◦ϕ.

(iii)
θ◦ϕ≤ϕ◦θ.

Theorem 3.22 *Let* A *be a congruence-permutable autometrized algebra. Then* A *is congruence-modular.
*

Proof.
Suppose A is a congruence-permutable. That is every pair of congruences on A are permutable.

Now to show that A is congruence-modular. To show that Con(A) is a modular.

Let $\Theta_{1},\;\Theta_{2},\;\Theta_{3}\in \mathrm{Con}\left(A\right)$ and $\Theta_{1}\subseteq \Theta_{3}$ . Clearly, $\Theta_{1}\vee \left(\Theta_{2}\wedge \Theta_{3}\right)\leq \left(\Theta_{1}\vee \Theta_{2}\right)\wedge \Theta_{3}$ .

To show that $\left(\Theta_{1}\vee \Theta_{2}\right)\wedge \Theta_{3}\leq \Theta_{1}\vee \left(\Theta_{2}\wedge \Theta_{3}\right)$ .

Let $\left(a,\;b\right)\in \left(\Theta_{1}\vee \Theta_{2}\right)\wedge \Theta_{3}$ . Therefore, $\left(a,\;b\right)\in \Theta_{1}\vee \Theta_{2}$ and $\left(a,\;b\right)\in \Theta_{3}$ . Since $\Theta_{1},\;\Theta_{2}$ are permutable; $\Theta_{1}\vee \Theta_{2}=\Theta_{1}◦\Theta_{2}$ . As a result, $\left(a,\;b\right)\in \Theta_{1}◦\Theta_{2}$ . There exists c∈A such that $\left(a,\;c\right)\in \Theta_{1}$ and $\left(c,\;b\right)\in \Theta_{2}$ . Since $\Theta_{1}\subseteq \Theta_{3}$ ; $\left(a,\;c\right)\in \Theta_{3}$ . Since $\Theta_{3}$ is a congruence relation; $\left(c,\;a\right)\in \Theta_{3}$ . Now we have $\left(c,\;a\right),\;\left(a,\;b\right)\in \Theta_{3}$ . Clearly, $\left(c,\;b\right)\in \Theta_{3}$ . As a result, $\left(c,\;b\right)\in \Theta_{2}\wedge \Theta_{3}$ .

Now we have $\left(a,\;c\right)\in \Theta_{1}$ and $\left(c,\;b\right)\in \Theta_{2}\wedge \Theta_{3}$ . Therefore, $\left(a,\;b\right)\in \Theta_{1}◦\left(\Theta_{2}\wedge \Theta_{3}\right)$ . Since A is a congruence-permutable; $\left(a,\;b\right)\in \Theta_{1}\vee \left(\Theta_{2}\wedge \Theta_{3}\right)$ . Therefore, $\left(\Theta_{1}\vee \Theta_{2}\right)\wedge \Theta_{3}\leq \Theta_{1}\vee \left(\Theta_{2}\wedge \Theta_{3}\right)$ . Consequently, $\Theta_{1}\vee \left(\Theta_{2}\wedge \Theta_{3}\right)=\left(\Theta_{1}\vee \Theta_{2}\right)\wedge \Theta_{3}$ . Hence, Con(A) is modular.

## 4. Homomorphism and isomorphism on autometrized algebra

In this section, we discuss the homomorphism, isomorphism, and correspondence theorems of autometrized algebra using the concept of congruence.

Theorem 4.1 *Let* A,B *be normal autometrized algebras. Let* f:A→B *be a homomorphism. Then the set*

kerf={(a,b)∈A×A|α(a)=α(b)}.

*is called kernel of the homomorphism* f .

Theorem 4.2 *Let* A,B *be normal autometrized algebras. Let* f:A→B *be a homomorphism. Then,* kerf *is a congruence on* A *. That is* kerf∈Con(A) .

Proof.
It is clear that kerf is an equivalence relation.

(i) Suppose (a,b),(c,d)∈kerf . So, f(a)=f(b) and f(c)=f(d) . Since f is a homomorphism; f(a+c)=f(a)+f(c)=f(b)+f(d)=f(b+d) . Therefore, (a+c,b+d)∈kerf

(ii) Suppose (a,b),(c,d)∈kerf . So, f(a)=f(b) and f(c)=f(d) . Since f is a homomorphism; f(a∗c)=f(a)∗f(c)=f(b)∗f(d)=f(b∗d) . Therefore, (a∗c,b∗d)∈kerf

(iii) Suppose (a,b)∈ker and x∗y≤a∗b . To show that $\left(x,\;y\right)\in \Theta_{1}◦\Theta_{2}$ . Clearly, f(a)=f(b) . Since f is a homomorphism; f(x∗y)≤f(a∗b) . As result, f(x)∗f(y)≤f(a)∗f(b) . Therefore, f(x)∗f(y)≤0 . So, f(x)=f(y) . Hence, (x,y)∈kerf .

Thus, kerf∈Con(A) .

Theorem 4.3 Let A be a normal autometrized algebra and Θ∈Con(A) . Define a map f:A→A/Θ by $f\left(a\right)=a/\Theta =\bar{a}$ . Then f is an epimorphism and kerf=Θ .

Proof.
Clearly Θ satisfies the condition. Hence A/Θ is a normal automertrized algebra. Clearly f is onto.

To show that f is homomorphism. Let a,b∈A .

$$\begin{array}{l} f\left(a+b\right)=\bar{a+b}=\bar{a}+\bar{b}=f\left(a\right)+f\left(b\right).\\ f\left(a*b\right)=\bar{a*b}=\bar{a}*\bar{b}=f\left(a\right)*f\left(b\right). \end{array}$$

Let a≤b . Then ∃x≥0 such that a+x=b . Therefore (a+x,b)∈Θ . Which implies that $\bar{a}\leq \bar{b}.$ That is; f(a)≤f(b) . Hence f is a homomorphism. So that f is an epimorphism from A to A/Θ . Consider;

$$\begin{array}{l} \ker f=\left\{\left(a,\;b\right)\in A\times A|f\left(a\right)=f\left(b\right)\right\}.\\ \;=\left\{\left(a,\;b\right)\in A\times A|\bar{a}\leq \bar{b}\right\}.\\ \;=\left\{\left(a,\;b\right)\in A\times A|a/\Theta =b/\Theta \right\}.\\ \;=\left\{a\in A|a*0\in \left(a,\;b\right)\in \Theta \right\}.\\ \;=\left(A\times A\right)\cap \Theta =\Theta . \end{array}$$

Theorem 4.4 *(Homomorphism theorem)* *Let A, B be normal autometrized algebras. Let f: A → B be a homomorphism. Then A/ *ker *f* ≅ *Imf. In particular if f is onto, then A/ *ker *f* ≅ *B.
*

Proof.
Let ker *f* = Θ. By the above theorem (4.3), the map

g:A→A/Θ is an epimorphism and kerg=Θ . Define a map h:A/Θ→Imf by $h\left(\bar{a}\right)=f\left(a\right)$ . Clearly, h is onto map. To show that h is well-defined and one to one. Suppose $\bar{a}=\bar{b}$ , then

$$\begin{array}{l} \bar{a}=\bar{b}\Leftrightarrow \left(a,b\right)\in \Theta .\\ \;\Leftrightarrow \left(a,b\right)\in \ker f.\\ \;\Leftrightarrow f\left(a\right)=f\left(b\right).\\ \;\Leftrightarrow h\left(\bar{a}\right)=h\left(\bar{b}\right). \end{array}$$

Therefore, h is well-defined and one to one.

Now to show that h is homomorphism. Let $\bar{a},\;\bar{b}\in A/\Theta$ . Consider;

$$\begin{array}{l} h\left(\bar{a}+\bar{b}\right)=h\left(\bar{a+b}\right).\\ \;=f\left(a+b\right).\\ \;=f\left(a\right)+f\left(b\right).\\ \;=h\left(\bar{a}\right)+h\left(\bar{b}\right). \end{array}$$

Again consider;

$$\begin{array}{l} \;h\left(\bar{a}*\bar{b}\right)=h\left(\bar{a*b}\right).\\ \;=f\left(a*b\right).\\ \;=f\left(a\right)*f\left(b\right).\\ \;=h\left(\bar{a}\right)*h\left(\bar{b}\right). \end{array}$$

Suppose let $\bar{a}\leq \bar{b}$ . Therefore ∃x≥0 such that (a+x,b)∈Θ . Hence,

$$\begin{array}{l} \;\left(a+x,\;b\right)\in \ker f.\\ \;\Rightarrow f\left(a+x\right)=f\left(b\right). \end{array}$$

Hence f(a)+f(x)=f(b) . Since $x\geq 0\Rightarrow f\left(x\right)\geq \bar{0}$ . Then f(a)≤f(b) . Therefore $h\left(\bar{a}\right)\leq h\left(\bar{b}\right)$ .

And thus h is a homomorphism. Therefore h is an isomorphism from A/Θ to Imf . That is A/Θ≅Imf . If f is onto map, then Imf=B . Hence A/Θ≅B .

Definition 4.5 *Let* A *and* B *be autometrized algebras and* θ,ϕ∈Con(A) *. Suppose* θ⊆ϕ .

*Define,* ϕ/θ={(a/θ,b/θ)∈A/θ×A/θ|(a,b)∈ϕ} *, and for any* (a/θ,b/θ),(c/θ,d/θ)∈ϕ/θ *;* a∗b/θ≤c∗d/θ ⇔ a∗b≤c∗d .

Theorem 4.6
*Let* A *be a normal autometrized algebra and* θ,ϕ∈Con(A) *. Suppose* θ⊆ϕ *. Then,
*

ϕ/θ∈Con(A/θ).

Proof.
First let us show that ϕ/θ is an equivalence relation on A.

1.
Reflexive : Let a/θ∈A/θ . Therefore, a∈A . Since ϕ is a congruence on A ; (a,a)∈ϕ . Hence, (a/θ,a/θ)∈ϕ/θ∀a∈A .
2.
Symmetric : Suppose (a/θ,b/θ)∈ϕ/θ . Then, (a,b)∈ϕ . Since ϕ is a congruence on A ; (b,a)∈ϕ . Hence, (b/θ,a/θ)∈ϕ/θ .
3.
Transitivity : Suppose (a/θ,b/θ),(b/θ,c/θ)∈ϕ/θ . Which implies that: (a,b),(b,c)∈ϕ . Thus, (a,c)∈ϕ . Therefore, (a/θ,c/θ)∈ϕ/θ . Hence, ϕ/θ is an equivalence relation.

To show that ϕ/θ is congruence on A. Let (a/θ,b/θ),(c/θ,d/θ)∈ϕ/θ . Then, (a,b),(c,d)∈ϕ .

(i) Since ϕ is a congruence; (a+c,b+d)∈ϕ . Since A/θ is a normal autometrized algebra; (a+c/θ,b+d/θ)∈ϕ/θ .
(ii) Since ϕ is a congruence; (a∗c,b∗d)∈ϕ . Since A/θ is a normal autometrized algebra; (a∗c/θ,b∗d/θ)∈ϕ/θ .
(iii) Let (a/θ,b/θ)∈ϕ/θ . Then (a,b)∈ϕ . Suppose x/θ∗y/θ≤a/θ∗b/θ . Since A/θ is normal autometrized algebra; x∗y/θ≤a∗b/θ . This implies that, ∃z≥0 such that (x∗y+z,a∗b)∈θ . Since θ⊆ϕ ; we obtain; (x∗y+z,a∗b)∈ϕ and implies x∗y/ϕ≤a∗b/ϕ . Therefore, x∗y≤a∗b . Since ϕ is a congruence on A ; (x,y)∈ϕ . Hence, (x/θ,y/θ)∈ϕ/θ .

Therefore, ϕ/θ∈Con(A) .

Theorem 4.7 **(Second Isomorphism Theorem)** *Let A be a normal autometrized algebras. Let θ,φ* ∈ *Con* ( *A*) *and θ* ⊆ *φ. Then*

$$\frac{A/\theta }{\phi /\theta }≅A/\phi .$$

Proof.
Define a map g:A/θ→A/ϕ by g(a/θ)=a/ϕ .

To show that g is well-defined.

Suppose; $\bar{a}=\bar{b}$ . That is;

$$\begin{array}{l} a/\theta =b/\theta \Leftrightarrow \left(a,b\right)\in \theta .\\ \;\Leftrightarrow \left(a,b\right)\in \phi .\left[\text{Since}\;\theta \subseteq \phi \right].\\ \;\Leftrightarrow a/\phi =b/\phi . \end{array}$$

Therefore, g(a/θ)=g(b/θ) . Hence, g is well-defined and one to one. Clearly g is onto map.

To show that g is a homomorphism.

Let a/θ,b/θ∈A/θ .

$$\begin{array}{l} g\left(a/\theta +b/\theta \right)=g\left(a+b/\theta \right).\\ \;=\left(a+b\right)/\phi .\\ \;=a/\phi +b/\phi .\\ \;=g\left(a/\theta \right)+g\left(b/\theta \right). \end{array}$$

Again consider;

$$\begin{array}{l} g\left(a/\theta *b/\theta \right)=g\left(a*b/\theta \right).\\ \;=\left(a*b\right)/\phi .\\ \;=a/\phi *b/\phi .\\ \;=g\left(a/\theta \right)*g\left(b/\theta \right). \end{array}$$

Suppose $\bar{a}\leq \bar{b}$ . That is; a/θ≤b/θ . Then ∃x≥0 such that (a+x,b)∈θ . Since θ⊆ϕ ; we obtain (a+x,b)∈ϕ . Therefore, a/ϕ≤b/ϕ . Hence g(a/θ)≤g(b/θ) .

Now let us consider the kernel of g .

$$\begin{array}{l} \ker g=\left\{(a/\theta ,\;b/\theta )|g\left(a/\theta \right)=g\left(b/\theta \right)\right\}.\\ \;=\left\{(a/\theta ,\;b/\theta )|a/\phi =b/\phi \right\}.\\ \;=\left\{(a/\theta ,\;b/\theta )|\left(a,b\right)\in \phi \right\}.\\ \;=\phi /\theta . \end{array}$$

Therefore, by first isomorphism theorem;

$$\frac{A/\theta }{\ker g}≅A/\phi .$$

Hence;

$\frac{A/\theta }{\phi /\theta }≅A/\phi .$

Theorem 4.8 *Let A be a normal autometrized algebras. Let B* ⊆ *A and θ* ∈ *Con*( *A*) *. Define*

$$B^{\theta }=\left\{a\in A|B\cap \left(a/\theta \right)\text{is}\;\text{non‐empty}\right\}.$$

Then, $B\subseteq B^{\theta }$ .

Proof.
Let b∈B . Since θ∈Con(A) implies that (b,b)∈θ . Then b∈b/θ . Therefore, b∈B∩(b/θ) . And thus, B∩(b/θ) is non-empty. So $b\in B^{\theta }$ . Hence, $B\subseteq B^{\theta }$ .

Definition 4.9 *Let A be a normal autometrized algebras. Let B be a normal subalgebra of A. Let θ* ∈ *Con*( *A*) *. Then, define, θ* | *B* = *θ* ∩ ( *B × B*).

Theorem 4.10 *Let A be a normal autometrized algebras. Let B be a normal subalgebra of A. Let θ ∈ Con*( *A*) *. Then, B ^θ^ is a normal subalgebra of A.
*

Proof.
Since 0 ∈ *B* and 0 ∈ 0/ *θ*; B ∩ (0/ *θ*) is non-empty. Hence 0 ∈ B *^θ^.*

Let *a*, *b* ∈ *B ^θ^.* Then, *B* ∩ ( *a*/ *θ*) and *B* ∩ ( *b*/ *θ*) are non-empty. Choose *x* ∈ *B* ∩ ( *a*/ *θ*) and *y* ∈ *B* ∩ ( *b*/ *θ*). Therefore; *x*, *y* ∈ *B* and *x* ∈ *a*/ *θ*, *y* ∈ *b*/ *θ.* Therefore, ( *x*, *a*), ( *y*, *b*) ∈ *θ.* Since *B* is a normal subalgebra of *A*;

$$x^{*}y,x+y\in B.$$ (2)

Since θ∈Con(A) ; (x+y,a+b)∈θ . Therefore,

x+y∈a+b/θ. (3)

By equations (2) and (3); we get: x+y∈B∩(a+b/θ) . Hence, B∩(a+b/θ) is non-empty. Therefore, $a+b\in B^{\theta }$ .

Similarly, Since θ∈Con(A) ; (x∗y,a∗b)∈θ . Therefore,

x∗y∈a∗b/θ. (4)

By equations (2) and (4); we get: x∗y∈B∩(a∗b/θ) . Hence, B∩(a∗b/θ) is non-empty. Therefore, $a*b\in B^{\theta }$ . Thus, $B^{\theta }$ is a subalgebra.

Now to show that $B^{\theta }$ is a normal subalgebra. Let $a,b\in B^{\theta }$ . Then, B∩(a/θ) and B∩(b/θ) are non-empty. Suppose a≤b . Since B is normal; ∃x≥0 such that b=a+x . Now to show that $x\in B^{\theta }$ . Clearly; x∈x/θ . Therefore, B∩(x/θ) is non-empty. This implies that $x\in B^{\theta }$ . Hence, $B^{\theta }$ is a normal subalgebra of A .

Theorem 4.11 *Let A be a normal autometrized algebras. Let B be a normal subalgebra of A. Let θ* ∈ *Con*( *A*) *. Then, θ* | *B is a congruence on B.
*

Proof.
Clearly, *θ* ∩ *B* × *B* is an equivalence relation on *B.*

Let ( *a*, *b*), ( *c*, *d*) ∈ *θ*/ *B* = *θ* ∩ ( *B* × *B*). Then, ( *a*, *b*), ( *c*, *d*) ∈ *B* × *B* and *θ.* Therefore *a*, *b*, *c*, *d* ∈ *B.*

(a) Since B is a normal subalgebra; *a* + *c*, *b* + *d* ∈ *B.* Therefore, ( *a* + *c*, *b* + *d*) ∈ *B* × *B.* And also *θ* is a congruence; ( *a* + *c*, *b* + *d*) ∈ *θ.* Hence, ( *a* + *c*, *b* + *d*) ∈ *θ* | *B.*
(b) Since B is a normal subalgebra; *a* ∗ *c*, *b* ∗ *d* ∈ *B.* Therefore, ( *a* ∗ *c*, *b* ∗ *d*) ∈ *B* × *B.* And also *θ* is a congruence; ( *a* ∗ *c*, *b* ∗ *d*) ∈ *θ.* Hence, ( *a* ∗ *c*, *b* ∗ *d*) ∈ *θ* | *B.*
(c) Suppose ( *a*, *b*) ∈ *θ* | *B* = *θ* ∩ *B* × *B* and *x* ∗ *y* ≤ *a* ∗ *b* ∀ *x*, *y*, *a*, *b* ∈ *B.* Therefore, ( *a*, *b*) ∈ *θ* and ( *a*, *b*) ∈ *B* × *B.* So *a*, *b* ∈ *B* and *a* ∗ *b* ∈ *B.* Since *θ* is congruence; implies ( *x*, *y*) ∈ *θ.* And clearly, ( *x*, *y*) ∈ *B* × *B.* Hence; ( *x*, *y*) ∈ *θ* ∩ *B* × *B.* And thus, *θ* | *B* is a congruence on *B.*

Theorem 4.12 **(The Third Isomorphism Theorem)** *Let A be a normal autometrized algebras. Let B be a normal subalgebra of A. Let θ* ∈ *Con*( *A*) *. Then,
*

$$\frac{B}{\theta |B}≅\frac{B^{\theta }}{\theta |B^{\theta }}.$$

Proof.
Clearly $\frac{B^{\theta }}{\theta |B^{\theta }}$ is a normal autometrized algebra.

Define a map $f:B\to \frac{B^{\theta }}{\theta |B^{\theta }}$ by $f\left(b\right)=\frac{b}{\theta |B^{\theta }}$ . To show that f is well-defined. Let $b_{1},b_{2}\in B$ . Suppose; $b_{1}=b_{2}$ . Therefore

$$\begin{array}{l} b_{1}=b_{2}\Rightarrow \frac{b_{1}}{\theta |B^{\theta }}=\frac{b_{2}}{\theta |B^{\theta }}.\\ \;\Rightarrow f\left(b_{1}\right)=f\left(b_{2}\right). \end{array}$$

Therefore, f is well-defined. To show that f is onto.

Let $\frac{c}{\theta |B^{\theta }}\in \frac{B^{\theta }}{\theta |B^{\theta }}$ . Therefore, $c\in B^{\theta }$ . Hence B∩(c/θ) is non-empty. Then ∃b∈B∩(c/θ) . Then b∈B and b∈c/θ . Since $B\subseteq B^{\theta }$ ; $b\in B^{\theta }$ . Therefore, $\left(b,c\right)\in B^{\theta }\times B^{\theta }$ and (b,c)∈θ . Clearly $\left(b,c\right)\in \theta \cap \left(B^{\theta }\times B^{\theta }\right)=\theta |B^{\theta }$ . Whence,

$$\frac{b}{\theta |B^{\theta }}=\frac{c}{\theta |B^{\theta }}.$$

Therefore, $f\left(b\right)=\frac{c}{\theta |B^{\theta }}$ . Hence, f is on to map.

To show that f is a homomorphism. Let a,b∈B . Consider;

$$\begin{array}{l} f\left(a+b\right)=\frac{a+b}{\theta |B^{\theta }}.\\ \;=\frac{a}{\theta |B^{\theta }}+\frac{b}{\theta |B^{\theta }}.\\ \;=f\left(a\right)+f\left(b\right). \end{array}$$

Again consider;

$$\begin{array}{l} f\left(a*b\right)=\frac{a*b}{\theta |B^{\theta }}.\\ \;=\frac{a}{\theta |B^{\theta }}*\frac{b}{\theta |B^{\theta }}.\\ \;=f\left(a\right)*f\left(b\right). \end{array}$$

Suppose a≤b . Then ∃x≥0 such that a+x=b . Therefore, $\frac{a+x}{\theta |B^{\theta }}=\frac{b}{\theta |B^{\theta }}$ . Which implies that $\left(a+x,b\right)\in \theta |B^{\theta }$ . Since $\frac{B^{\theta }}{\theta |B^{\theta }}$ is normal implies that $\frac{a}{\theta |B^{\theta }}\leq \frac{b}{\theta |B^{\theta }}$ . Thus, f(a)≤f(b) . Hence, f is a homomorphism.

Now consider the kernel of f . Therefore,

$$\begin{array}{l} \ker f=\left\{\left(a,\;b\right)\in B\times B|f\left(a\right)=f\left(b\right)\right\}.\\ \;=\left\{\left(a,\;b\right)\in B\times B|\frac{a}{\theta |B^{\theta }}=\frac{b}{\theta |B^{\theta }}\right\}.\\ \;=\left\{\left(a,\;b\right)\in B\times B|\left(a,b\right)\in \theta |B^{\theta }\right\}.\\ \;=\left\{\left(a,\;b\right)\in B\times B|\left(a,b\right)\in \theta \cap B^{\theta }\times B^{\theta }\right\}.\\ \;=\left(B\times B\right)\cap \theta \cap \left(B^{\theta }\times B^{\theta }\right).\\ \;=\left(B\times B\right)\cap \theta .\left[\text{Since}\;B\subseteq \;B^{\theta }\right]\\ \;=\theta |B. \end{array}$$

Therefore, by first isomorphism theorem;

$$\frac{B}{\ker f}≅\frac{B^{\theta }}{\theta |B^{\theta }}.$$

Hence;

$\frac{B}{\theta |B}≅\frac{B^{\theta }}{\theta |B^{\theta }}.$

Theorem 4.13 **(Correspondence Theorem)** *Let A be a normal autometrized algebra. Let θ* ∈ *Con*( *A*). *Then* [ *θ*, ∇ *_A_*] *is lattice isomorphic to Con* ( *A*/ *θ*).

Proof.
We know that I(A) and *Con*( *A*) are lattices.

We know that by theorem (2.10), I(A) and *Con*( *A*) are in one to one correspondence. Therefore, [ *θ*, ∇ *_A_*] is an interval in *Con*( *A*). Therefore, [ *θ*, ∇ *_A_*] is a sublattice of *Con*( *A*). We know that *Con*( *A*/ *θ*) is a lattice.

Define a map *f*: [ *θ*, ∇ *_A_*] → *Con*( *A*/ *θ*) by *f*( *ϕ*) = *ϕ*/ *θ.* Since *ϕ* ∈ [ *θ*, ∇ *_A_*]; implies that *θ* ⊆ *ϕ* ⊆ ∇ *_A_.* Therefore, by theorem (4.6); *ϕ*/ *θ* ∈ *Con*( *A*/ *θ*).

To show that *f* is well-defined and one to one.

Let *ϕ*, *ψ* ∈ [ *θ*, ∇ *_A_*]. Therefore, *θ* ⊆ ϕ, *ψ* ⊆ ∇ *_A_.*

Suppose *ϕ* = *ψ.* Therefore, *ϕ*/ *θ* = *ϕ*/ *θ.* Which implies that *f*( *ϕ*) = *f*( *ψ*). Hence, f is well-defined.

Suppose *f*( *ϕ*) = *f*( *ψ*). Therefore, *ϕ*/ *θ* = *ψ*/ *θ.*

Let ( *a*, *b*) ∈ *ϕ.* Therefore,

$$\begin{array}{l} \left(a/\theta ,b/\theta \right)\in \phi /\theta .\\ \left(a/\theta ,b/\theta \right)\in \psi /\theta .\\ \;\left(a,b\right)\in \psi .\\ \;\phi \subseteq \psi . \end{array}$$

And similarly, let (a,b)∈ψ . Therefore,

$$\begin{array}{l} \left(a/\theta ,b/\theta \right)\in \psi /\theta .\\ \left(a/\theta ,b/\theta \right)\in \phi /\theta .\\ \;\left(a,b\right)\in \phi .\\ \;\psi \subseteq \phi . \end{array}$$

Whence, ϕ=ψ . Hence, f is one to one.

To show that f is on to. Let $\theta_{1}\in \mathrm{Con}\left(A/\theta \right)$ .

Define

$$\begin{array}{l} \theta_{2}=\left\{(a,b)|\frac{a/\theta }{\theta_{1}}=\frac{b/\theta }{\theta_{1}}\right\}.\\ \theta_{2}=\left\{(a,b)|\left(a/\theta ,b/\theta \right)\in \theta_{1}\right\} \end{array}$$

Now, to show that $\theta_{2}\in \mathrm{Con}\left(A\right).$

Let $\left(a,b\right),\left(c,d\right)\in \theta_{2}$ . So $\left(a/\theta ,b/\theta \right),\left(c/\theta ,d/\theta \right)\in \theta_{1}$ . Since $\theta_{1}\in \mathrm{Con}\left(A/\theta \right)$ ; $\left(a/\theta +c/\theta ,b/\theta +d/\theta \right)\in \theta_{1}$ and $\left(a/\theta *c/\theta ,b/\theta *d/\theta \right)\in \theta_{1}$ . Which implies that $\left(a+c,b+d\right)\in \theta_{2}$ and $\left(a*c,b*d\right)\in \theta_{2}$ .

Let $\left(a,b\right)\in \theta_{2}$ and x∗y≤a∗b . So $\left(a/\theta ,b/\theta \right)\in \theta_{1}$ . By definition (4.5); x∗y/θ≤a∗b/θ ⇔ x∗y≤a∗b . Since $\theta_{1}$ is congruence; $\left(x/\theta ,y/\theta \right)\in \theta_{1}$ . Whence, $\left(x,y\right)\in \theta_{2}$ . Hence, $\theta_{2}\in \mathrm{Con}\left(A\right)$ .

Let (a,b)∈θ . Therefore,

$$\begin{array}{l} a/\theta =b/\theta .\\ \frac{a/\theta }{\theta_{1}}=\frac{b/\theta }{\theta_{1}}.\\ \left(a,b\right)\in \theta_{2}. \end{array}$$

Therefore, $\theta \subseteq \theta_{2}\subseteq \nabla_{A}$ . Hence $\theta_{2}\in \left[\theta ,\nabla_{A}\right]$ .

Now to show that $f\left(\theta_{2}\right)=\theta_{1}$ . To show that $\theta_{2}/\theta =\theta_{1}$ . Let $\left(a/\theta ,b/\theta \right)\in \theta_{2}/\theta$ .

$$\begin{array}{l} \;\left(a,b\right)\in \theta_{2}.\\ \;\frac{a/\theta }{\theta_{1}}=\frac{b/\theta }{\theta_{1}}.\\ \left(a/\theta ,b/\theta \right)\in \theta_{1}. \end{array}$$

Therefore, $\theta_{2}/\theta \subseteq \theta_{1}$ .

Let $\left(a/\theta ,b/\theta \right)\in \theta_{1}$ . Therefore,

$$\begin{array}{l} \;\frac{a/\theta }{\theta_{1}}=\frac{b/\theta }{\theta_{1}}.\\ \;\left(a,b\right)\in \theta_{2}.\\ \left(a/\theta ,b/\theta \right)\in \theta_{2}/\theta . \end{array}$$

Therefore, $\theta_{1}\subseteq \theta_{2}/\theta$ . Hence, $\theta_{2}/\theta =\theta_{1}$ . Thus, $f\left(\theta_{2}\right)=\theta_{1}$ . Therefore, f is onto.To show that $\theta_{1}\subseteq \theta_{2}\Leftrightarrow f\left(\theta_{1}\right)\subseteq f\left(\theta_{2}\right).$

Let $\theta_{1},\theta_{2}\in \left[\theta ,\nabla_{A}\right]$ . Suppose $\theta_{1}\subseteq \theta_{2}$ .

Let $\left(a/\theta ,b/\theta \right)\in \theta_{1}/\theta$ .

$$\begin{array}{l} \;\left(a,b\right)\in \theta_{1}.\\ \;\left(a,b\right)\in \theta_{2}.\\ \left(a/\theta ,b/\theta \right)\in \theta_{2}/\theta . \end{array}$$

Therefore, $\theta_{1}/\theta \subseteq \theta_{2}/\theta$ . Hence, $f\left(\theta_{1}\right)\subseteq f\left(\theta_{2}\right)$ .

Conversely, suppose $f\left(\theta_{1}\right)\subseteq f\left(\theta_{2}\right)$ . Therefore, $\theta_{1}/\theta \subseteq \theta_{2}/\theta$ .

Let $\left(a,b\right)\in \theta_{1}$ .

$$\begin{array}{l} \left(a/\theta ,b/\theta \right)\in \theta_{1}/\theta .\\ \left(a/\theta ,b/\theta \right)\in \theta_{2}/\theta .\\ \;\left(a,b\right)\in \theta_{2}. \end{array}$$

Therefore, $\theta_{1}\subseteq \theta_{2}$ . Whence, $\theta_{1}\subseteq \theta_{2}\Leftrightarrow f\left(\theta_{1}\right)\subseteq f\left(\theta_{2}\right)$ . Therefore, f is an ordered isomorphism of $\left[\theta ,\nabla_{A}\right]$ onto Con(A/θ) . Hence, $\left[\theta ,\nabla_{A}\right]≅\mathrm{Con}\left(A/\theta \right)$ .

## 5. Conclusion

This paper presented the study of congruence relation on autometrized algebras. We introduced in normal autometrized algebra satisfying some conditions the set of all congruence relations form a complete sublattice of the set of all equivalence relations. We also examined that a congruence-permutable autometrized algebra is congruence-modular. We explored several fundamental properties related to congruence relations. Additionally, we introduced the ker of a homomorphism and showed that it is a congruence relation. We also examined the homomorphism, and isomorphism correspondence theorems of autometrized algebra by using congruency.

## Author contributions

All the authors are contributed equally in this manuscript and also both authors read and approved the final manuscript.

## Ethics and consent

Ethics and consent were not required.

## Data Availability

No data are associated with this article.
