# Supplementary material for: Congruency, Homomorphism  and Isomorphism on Autometrized Algebras
Source: F1000Res. 2025 Aug 22;14:22. Originally published 2025 Jan 3. [Version 2] doi: 10.12688/f1000research.159591.2 (PMC12461190; doi:10.12688/f1000research.159591.2)
Supplement: Peer review history

# Reviewer report: Reviewer response for version 2

**DOI:** 10.5256/f1000research.186585.r408526

**Published:** 2025-09-24

**Authors:** Alachew Amaneh Mechderso

**Affiliations:** 1 University of KabriDahar, Kabri Dahar, Ethiopia

The paper titled *"Congruency, Homomorphism and Isomorphism on Autometrized Algebra"* delivers its content in a clear, organized, and coherent way. The authors effectively break down and explain complex algebraic concepts, using precise language that enhances understanding. Their structured presentation makes the material both informative and approachable for readers with prior knowledge in the field.

Is the work clearly and accurately presented and does it cite the current literature?

Yes

If applicable, is the statistical analysis and its interpretation appropriate?

Yes

Are all the source data underlying the results available to ensure full reproducibility?

Yes

Is the study design appropriate and is the work technically sound?

Yes

Are the conclusions drawn adequately supported by the results?

Yes

Are sufficient details of methods and analysis provided to allow replication by others?

Yes

Reviewer Expertise:

fuzzy  Algebra, Fuzzy Uniform Toplogy

I confirm that I have read this submission and believe that I have an appropriate level of expertise to confirm that it is of an acceptable scientific standard.

# Reviewer report: Reviewer response for version 1

**DOI:** 10.5256/f1000research.175346.r358302

**Published:** 2025-06-20

**Authors:** Sileshe Gone Korma

**Affiliations:** 1 Hawassa University, Hawassa, Ethiopia

This paper investigates the structure and properties of congruence relations in autometrized algebras. It establishes that, under specific conditions, the congruence relations in a normal autometrized algebra form a complete sublattice within the equivalence relations. The study also shows that congruence-permutable autometrized algebras exhibit congruence-modularity. Key fundamental properties of congruence relations are explored, including the introduction of the kernel of a homomorphism as a congruence relation. Additionally, the paper examines homomorphism, isomorphism, and correspondence theorems in the context of autometrized algebra, utilizing the framework of congruence.

 Authors should include the following corrections

**Comments**

1. Section 1 Defnition 2.4 (ii); ( *B, ≤)*  is subposet of A , and ≤ is translation invariant, that is, *∀a, b, c ∈ A; a ≤ b ⇒ a+c ≤ b+c*
2. Section 4

1. Theorem 4.1; line 2; *f(a) = f(b)* .
2. Theorem 4.2; item (iii); line 1 and 2; make Ker f and make (x, y) *∈*ker *f* .
3. Theorem 4.3; line 12; make  ker *f=* *a, b* *∈A×A|* *a, b* *∈θ* *.*
4. Theorem 4.7; line 1 and 2; substitute *φ by ϕ* .
5. Theorem 4.10; equation 2; make x*y .
6. Theorem 4.13; line 10; *ϕ* *θ* *=* *ψ* *θ*  .

1. Section 5

Conclusion; line 4; make kernel.

Is the work clearly and accurately presented and does it cite the current literature?

Yes

If applicable, is the statistical analysis and its interpretation appropriate?

Not applicable

Are all the source data underlying the results available to ensure full reproducibility?

No source data required

Is the study design appropriate and is the work technically sound?

Yes

Are the conclusions drawn adequately supported by the results?

Yes

Are sufficient details of methods and analysis provided to allow replication by others?

Yes

Reviewer Expertise:

Algebra specifically in lattices and fuzzy algebra

I confirm that I have read this submission and believe that I have an appropriate level of expertise to confirm that it is of an acceptable scientific standard.

# Response

**Published:** 2025-08-13

**Authors:** Gebrie Yeshiwas Tilahun

**Affiliations:** Mathematics, Assosa University, Asosa, Benishangul-Gumuz, Ethiopia

We incorporated all suggested comments and submitted the corrected version.

# Reviewer report: Reviewer response for version 1

**DOI:** 10.5256/f1000research.175346.r362466

**Published:** 2025-02-25

**Authors:** Abdelmohsen Badawy

**Affiliations:** 1 Mathematics, Faculty of science, Tanta University, Tanta, Gharbia Governorate, Egypt

Dear Professor

 Thank you. I go through the paper, it has small new results, it needs a major improve English, improve the presentation of the paper, and the author must add the basic concepts which are needed as equivalent class, quotient set and explain how the set Eq of all equivalent relation forms a lattice. Also Must construct example to explore the results. I listed some remarks in the attached report.

Is the work clearly and accurately presented and does it cite the current literature?

Partly

If applicable, is the statistical analysis and its interpretation appropriate?

Partly

Are all the source data underlying the results available to ensure full reproducibility?

Partly

Is the study design appropriate and is the work technically sound?

Partly

Are the conclusions drawn adequately supported by the results?

Partly

Are sufficient details of methods and analysis provided to allow replication by others?

Partly

Reviewer Expertise:

Algebra- Lattice Theory - MS-algebras

I confirm that I have read this submission and believe that I have an appropriate level of expertise to confirm that it is of an acceptable scientific standard, however I have significant reservations, as outlined above.

# Response

**Published:** 2025-08-13

**Authors:** Gebrie Yeshiwas Tilahun

**Affiliations:** Mathematics, Assosa University, Asosa, Benishangul-Gumuz, Ethiopia

We incorporated all comments as the reviewer's suggestions. Such as:

 1. the definition of as equivalent class and quotient set are given. Also, they are illustrated by examples.

 2. the proof of the set of all equivalent relation forms a complete lattice is incorporated.

 3. English and the presentation of the paper are improved.
